# Supplementary material for: Novel Driver Strength Index highlights important cancer genes in TCGA PanCanAtlas patients
Source: PeerJ. 2022 Aug 11;10:e13860. doi: 10.7717/peerj.13860 (PMC9375969; doi:10.7717/peerj.13860)
Supplement: Supplemental Information 2 [file peerj-10-13860-s002.zip › COHORTS/genes plots/stage/2021_8_16_14_9_distribution_stage_IV.pdf]

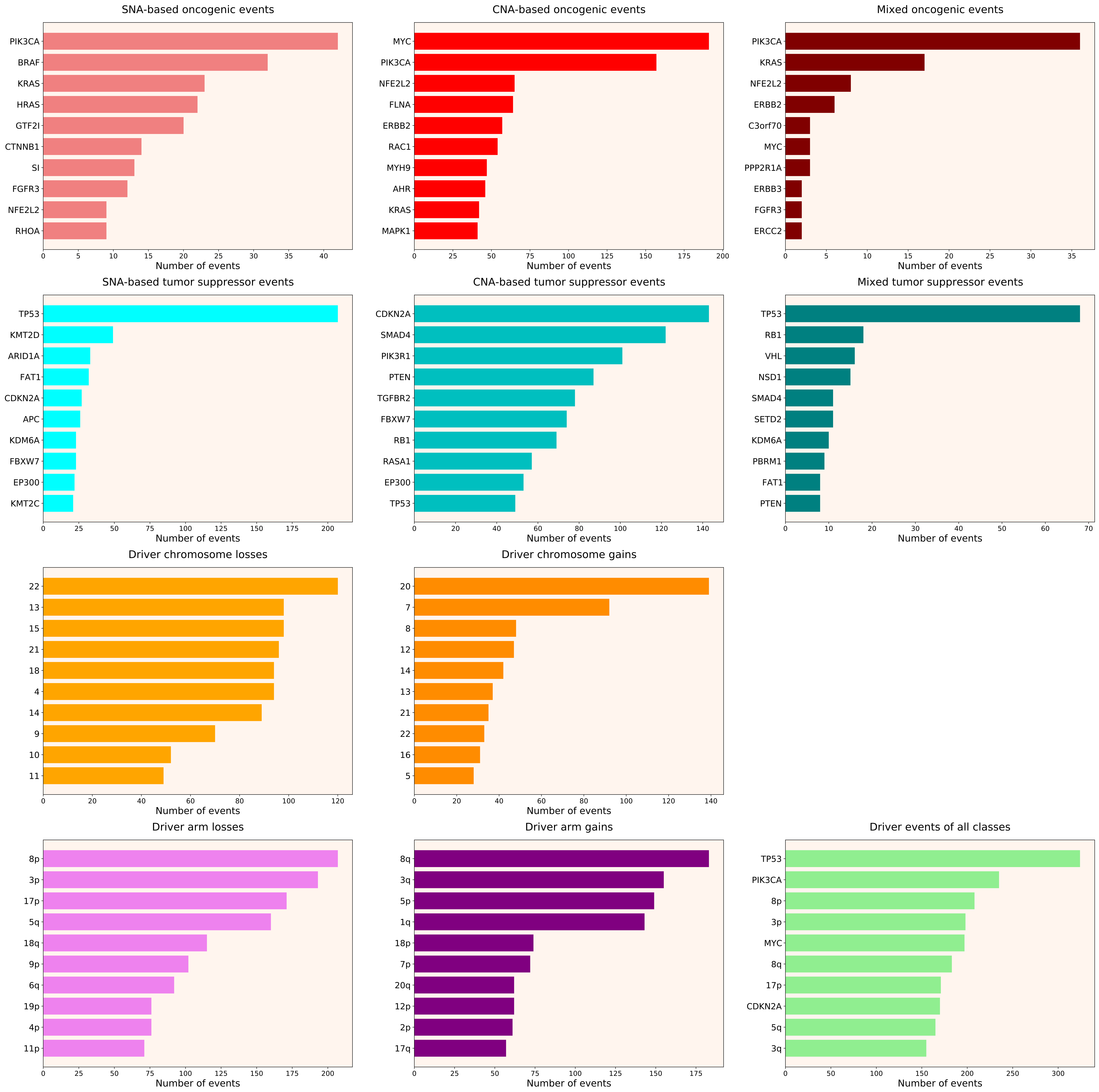

SNA-based oncogenic events
PIK3CA
BRAF
KRAS
HRAS
GTF2I
CTNNB1
SI
FGFR3
NFE2L2
RHOA
Number of events
CNA-based oncogenic events
MYC
PIK3CA
NFE2L2
FLNA
ERBB2
RAC1
MYH9
AHR
KRAS
MAPK1
Number of events
Mixed oncogenic events
PIK3CA
KRAS
NFE2L2
ERBB2
C3orf70
MYC
PPP2R1A
ERBB3
FGFR3
ERCC2
Number of events
SNA-based tumor suppressor events
TP53
KMT2D
ARID1A
FAT1
CDKN2A
APC
KDM6A
FBXW7
EP300
KMT2C
Number of events
CNA-based tumor suppressor events
CDKN2A
SMAD4
PIK3R1
PTEN
TGFBR2
FBXW7
RB1
RASA1
EP300
TP53
Number of events
Mixed tumor suppressor events
TP53
RB1
VHL
NSD1
SMAD4
SETD2
KDM6A
PBRM1
FAT1
PTEN
Number of events
Driver chromosome losses
22
13
15
21
18
4
14
9
10
11
Number of events
Driver chromosome gains
20
7
8
12
14
13
21
22
16
5
Number of events
Driver arm losses
8p
3p
17p
5q
18q
9p
6q
19p
4p
11p
Number of events
Driver arm gains
8q
3q
5p
1q
18p
7p
20q
12p
2p
17q
Number of events
Driver events of all classes
TP53
PIK3CA
8p
3p
MYC
8q
17p
CDKN2A
5q
3q
Number of events
